# The Causation of Disease

**Published:** 1889-03

**Authors:** 


*The Causation of Disease.* By HARRY CAMPBELL, M.D. Pp. 308. London: H. K. LEWIS. 1889.

At the outset one is not prepossessed in favour of a writer dealing with advanced philosophy who quotes (p. 52) Tennyson as saying: "In the spring a livelier iris brightens on the wing of dove," instead of "changes on the burnished dove;" who uses throughout the work "ante-partem" to express what we mean by "ante-partum;" and who makes numerous similar mistakes which can scarcely be misprints. "Creighton Brown" for Crichton Browne (p. 52) is an error as much in courtesy as in spelling.

On the first page we are startled with the query "What is the cause of O uniting, or, in simpler language, *why* does O unite with H in the proportion to form water?" It is not oxygen and hydrogen, but O and H. Environment is E, structure is S; and E and S play very important parts in this work. Medical observations are not numerous, but some of them are worthy of notice. For instance, discussing whether there is a monthly rhythm in the male sex analogous to that in the female. He says: "I have met with a case of monthly-bleeding piles in an elderly gentleman; and it is a very interesting fact that when, at the age of sixty-six, the hæmorrhagic flux became irregular, this gentleman experienced many of the symptoms which women complain of at the climacteric—he became emotional, and, among other things, suffered from very marked flushings, headache and backache, and a symptom which I have never yet found absent from among these menopausic *(sic)* patients who seek medical relief—*soreness of the scalp.*"

We have read the book from beginning to end, not without interest, occasionally with advantage to our mind, but sometimes to the detriment of our temper. The author has an original and philosophic bent, and presents many facts in suggestive lights and sequences. But a more intimate acquaintance with Bain and Mill and Spencer and Darwin than our author possesses is necessary for a man who enters on a philosophic disquisition on the causation of anything biological; and an extended clinical experience in addition is essential for the satisfactory consideration of the causation of disease in particular. The work is suggestive and interesting: if Dr. Harry Campbell waits for twenty years of clinical experience, and in the meantime studies philosophy in general and the logic of causation in particular, he may provide us with a work of some value.

